# Ab-externo drainage with continuous anterior chamber infusion for non-resolving exudative retinal detachment: a case report

**DOI:** 10.1186/s12886-020-01589-5

**Published:** 2020-08-28

**Authors:** Matthew P. Simunovic, Emily H. Shao, Perach Osaadon

**Affiliations:** 1 Save Sight Institute, University of Sydney, 8 Macquarie Street, Sydney, NSW 2000 Australia; 2 Sydney Eye Hospital, 8 Macquarie Street, Sydney, NSW 2000 Australia; 3 Nuffield Laboratory of Ophthalmology, John Radcliffe Hospital, Oxford, OX3 9DU UK

**Keywords:** Exudative retinal detachment, Retinal detachment, Sub-retinal fluid drainage, Vogt-Koyanagi-Hara syndrome

## Abstract

**Background:**

Drainage of exudative retinal detachment may be necessary for either therapeutic or diagnostic purposes (or both). Here, we describe an external drainage technique for non-resolving vision-threatening exudative retinal detachment which combines the advantages of internal drainage (widefield viewing and intraocular pressure control using continuous anterior chamber infusion) with those of external drainage (drainage of sub-retinal fluid without vitrectomy).

**Case presentation:**

To illustrate this technique, we present a 13-year-old girl with macula-off exudative retinal detachment secondary to Vogt-Koyanagi-Harada syndrome, which was unresponsive to aggressive medical management. External drainage was undertaken using widefield viewing and chandelier illumination. Intraocular pressure was maintained with an anterior chamber infusion. Near-complete drainage of sub-retinal fluid was achieved, and retinal reattachment was maintained at 6 months postoperatively, with a corresponding improvement in visual acuity from 20/63 to 20/40.

**Conclusions:**

External drainage under chandelier-assisted viewing at the surgical microscope with anterior chamber infusion offers the ergonomic and optical advantages of the surgical microscope and widefield visualisation, continuous IOP control and drainage of sub-retinal fluid without the need for pars plana vitrectomy.

## Background

Exudative retinal detachment is primarily managed medically; however, surgical drainage may be indicated in cases where less invasive procedures, such as systemic therapy, laser and peri−/intra-ocular medical approaches, fail. Surgical approaches can be divided into internal drainage — where fluid is aspirated via a retinotomy following trans-pars plana vitrectomy (TPPV) — and external drainage, where fluid is removed via a trans-chorioscleral route (with, or without peritomy) [1]. The external approach may be preferable to the internal approach in young, phakic patients for several reasons. First, the internal approach generally requires the creation of a posterior vitreous detachment, which in turn confers a risk of inducing iatrogenic breaks. Second, it risks the development of proliferative vitreoretinopathy (PVR). Third, it can rarely cause iatrogenic cataract through unwanted lens touch. However, it is argued that internal surgery provides a superior view of the posterior segment intra-operatively, carries a lower risk of subretinal haemorrhage (which can occur in up to 28% of external needle drainage procedures) [2, 3], allows easier access to sub-retinal fluid (which in the context of ERD loculates posteriorly with the patient supine on the operating table) and — with modern surgical systems — avoids the hypotony which typically occurs following external drainage [4].

Here, we describe a technique which combines the advantages of the internal approach (widefield view at the surgical microscope, control of intraocular pressure via continuous infusion) and the external approach (drainage of sub-retinal fluid without the need for vitrectomy or the creation of a drainage retinotomy) which may be suitable for the majority of patients with exudative retinal detachment (ERD) who cannot be adequately managed with non-surgical measures alone.

## Case presentation

**Fig. 1:**
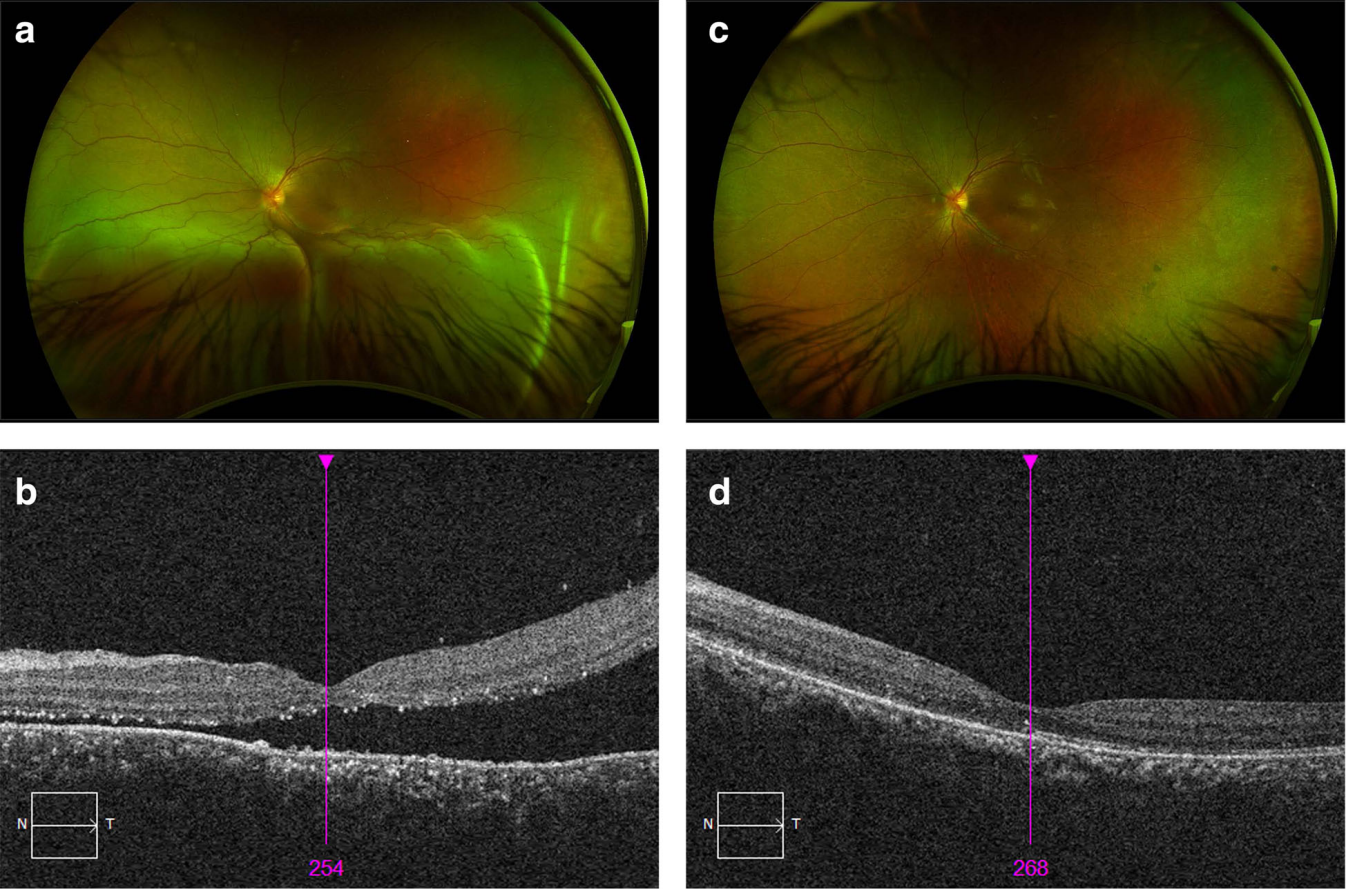
(**a**) Preoperative widefield fundus photograph demonstrating an inferior exudative retinal detachment involving the macula, confirmed on optical coherence tomography (**b**). (**c**) Post-operative fundus appearance at 2-months, demonstrating retinal reattachment, confirmed on optical coherence tomography (**d**)

A 13-year-old girl of South-East Asian ethnicity was referred to the vitreoretinal service at Sydney Eye Hospital for possible surgical management of a left bullous ERD involving the macula (with the eye in the primary position whilst upright). She had initially presented 5 months earlier with vision loss from a right bullous ERD involving the macula and a left limited inferior ERD. After extensive ocular and systemic workup, she was diagnosed with incomplete Vogt-Koyanagi-Harada syndrome [5]. Despite treatment with high-dose intravenous corticosteroid, azathioprine and cyclosporin, her condition worsened with total ERD in the right eye and inferior ERD in the left. She underwent right conventional vitrectomy for total non-resolving ERD under the care of another surgeon, though no breaks were identified at the time of surgery: silicone oil was used as the vitreous substitute. Her vision postoperatively was right 20/200, and despite the subsequent addition of adalimumab, her left ERD progressed to involve the macula (Fig. 1a & b) with a reduction in visual acuity from 20/20 to 20/63. Given the progress of the right eye, a decision was made to drain the left ERD surgically. Institutional approval was not required for this intervention, which was conducted after gaining the informed and unanimous consent of the patient and her parents.

As no breaks were identified on careful preoperative examination, an external drainage approach was planned. The case commenced with a 360-degree peritomy; the four rectus muscles were isolated in turn and slung with 4–0 silk sutures to gain external control of the eye (video). An inferotemporal paracentesis was created with a 15-degree blade: a 25G port was then introduced into the anterior chamber via a modified Seldinger technique [6], in which a port is slid over the top of a trocar placed within the paracentesis wound (video). An infusion line and cannula were inserted into the port and switched on (video). A trans-pars plana port was subsequently placed 4 mm posterior to the corneoscleral limbus superonasally and a chandelier used to provide intraocular illumination (video). The peripheral ocular fundus was carefully inspected under widefield view with a ReSite viewing system (Carl Zeiss AG, Oberkochen, Germany) using a cryotherapy probe to indent the peripheral retina: no retinal breaks were present. An external drainage and depression device (“EDD”, Vortex Surgical, MO, USA), which features an extendable guarded 28G drainage needle, was connected to a 1 mL syringe and then used to indent the peripheral retina under direct visualisation. Once the resulting indent was observed in the inferior mid-peripheral retina (video), the intraocular pressure was increased to 60 mmHg to facilitate collapse of the choroidal vascular bed (and to promote hemostasis in the event of choroidal/sub-retinal hemorrhage) and the drainage needle was then extended bevel-side down. Subretinal fluid was slowly drained actively by an assistant: 1.2 mL was drained in total which was sent for laboratory analysis, including microscopy, culture, sensitivity (negative) and cytopathology (no abnormal cells identified). The needle was removed from the eye prior to the retina coming into contact with the drainage needle (video). The intraocular pressure was lowered to 35 mmHg, and the superonasal port was then removed and the site closed with 8–0 vicryl and the limbal infusion cannula with port was removed. The paracentesis wound was hydrated and self-sealed using balanced salt solution on a Rycroft cannula. The conjunctiva was closed with interrupted 8–0 vicryl sutures.


**Additional file 1**

The patient’s retina was flat and attached on day one. She was maintained on adalimumab and her retina remained flat and attached at 6 months, with an improvement in visual acuity to 20/40 (Fig. 1c & d).

## Discussion & conclusion

Exudative retinal detachment may occur secondary to a variety of disease processes, including vascular diseases, inflammatory disease and neoplastic processes. In the majority of cases, non-surgical approaches are appropriate. However, surgical reattachment may be indicated for vision-threatening ERD which does not resolve with such measures and/or for diagnostic purposes. In our case, we presume that retinal pigment epithelium-facilitated fluid reabsorption mechanisms were impaired pre-operatively, but were sufficient to maintain reattachment post-operatively.

Although a conventional vitrectomy approach can be considered in the management of ERD, it is associated with several disadvantages. Internal drainage following vitrectomy necessitates retinotomy and may also be associated with iatrogenic retinal tears, both of which may facilitate the development of subsequent rhegmatogenous retinal detachment and PVR. Another significant disadvantage in phakic patients is the potential development of cataract.

The surgical approach described above is a refinement and combination of several techniques. Recent advances in viewing and endo-illumination systems have resulted in increased interest in — and reports of — non-vitrectomy vitreoretinal surgery conducted with the aid of a surgical microscope [7]. Anterior chamber infusion is a suitable, but often overlooked means of continuous intraocular pressure maintenance in phakic patients which obviates the creation of an additional sclerotomy [8]. It is attractive because the infusion line is easily visualized; however, there remains the possibility unwanted lens touch, and it may rarely be ineffective if there is blockage of flow (e.g. in the presence of dense cyclitic membranes). External drainage with active aspiration is most commonly performed in the context of scleral buckling surgery for rhegmatogenous retinal detachment [2, 3], and the extendable needle used in our case offers the advantage of allowing safe indentation prior to the introduction of a guarded needle in known orientation.

In summary, external drainage under chandelier-assisted direct view at the surgical microscope with anterior chamber infusion offers the ergonomic and optical advantages of the surgical microscope and widefield viewing system, continuous control of IOP and near-complete drainage of sub-retinal fluid which can be aspirated via syringe and sent for laboratory testing.

## Data Availability

All relevant clinical details are included in the accompanying article, images and video.

## Abbreviations

ERD: Exudative retinal detachment
IOP: Intraocular pressure
PVR: Proliferative vitreoretinopathy
TPPV: Trans-pars plana vitrectomy
